# A bibliometric analysis of global research output on network meta-analysis

**DOI:** 10.1186/s12911-021-01470-5

**Published:** 2021-05-03

**Authors:** Jiyuan Shi, Ya Gao, Liu Ming, Kelu Yang, Yue Sun, Ji Chen, Shuzhen Shi, Jie Geng, Lun Li, Jiarui Wu, Jinhui Tian

**Affiliations:** 1 Evidence-Based Nursing Centre, School of Nursing, Lanzhou University, Lanzhou City, China; 2 Evidence-Based Medicine Center, School of Basic Medical Sciences, Lanzhou University, Lanzhou City, China; 3 School of Nursing, Peking University, Beijing City, China; 4 Mianyang hospital of traditional Chinese medicine, Mianyang City, China; 5 Department of General Surgery, The Second Hospital of Lanzhou University, Lanzhou City, China; 6 Second Xiangya Hospital, Central South University, Changsha City, China; 7 Department of Clinical Chinese Pharmacy, School of Chinese Materia Medica, Beijing University of Chinese Medicine, Beijing City, China; 8 Key Laboratory of Evidence-Based Medicine and Knowledge Translation of Gansu Province, Lanzhou City, China

**Keywords:** Bibliometric analysis, Network meta-analyses, Development trends, Hotspots, Web of Science, CiteSpace

## Abstract

**Background:**

Network meta-analysis (NMA) has been widely used in the field of medicine and health, but the research topics and development trends are still unclear. This study aimed to identify the cooperation of countries and institutes and explore the hot topics and future prospects in the field of NMA.

**Methods:**

Data of publications were downloaded from the Web of Science Core Collection. We used CiteSpace V, HistCite 2.1, and Excel 2016 to analyze literature information, including years, journals, countries, institutes, authors, keywords, and co-cited references.

**Results:**

NMA research developed gradually before 2010 and rapidly in the following years. 2846 NMA studies were published in 771 journals in six languages. The *PLoS One* (110, 3.9%) was the most productive journal, and *N Engl J Med* (5904 co-citations) was the most co-cited journal. The most productive country was the United States (889, 31%) and the most productive institute was the University of Bristol (113, 4.0%). The active collaborations were observed between developed countries and between productive institutes. Of the top 10 authors, four were from the UK, and among the top 10 co-cited authors, six were from the UK. Randomized evidence, oral anti-diabetic drugs, coronary artery bypass, certolizumab pegol, non-valvular atrial fibrillation, and second-line antihyperglycemic therapy were the hot topics in this field.

**Conclusions:**

NMA studies have significantly increased over the past decade, especially from 2015 to 2017. Compared with developing countries, developed countries have contributed more to these publications and have closer cooperation, indicating that cooperation between developed and developing countries should be further strengthened. The treatment of diabetes, cardiovascular diseases, and immune rheumatism are the main hot topics.

## Background

Network meta-analysis (NMA), also known as mixed treatment comparisons, is an extension of the pairwise meta-analysis method for integrating data from trials to compare at least two competing healthcare interventions [1–4]. Compared with traditional meta-analysis, NMAs allow for the synthesis and comparison of evidence from multiple interventions, including direct and indirect evidence, to provide more accurate estimates of treatment outcomes, even though head-to-head trials may be lacking [5–7]. NMAs can also generate a relative ranking of all interventions related to the outcome to provide valuable information for patients, practitioners, and decision-makers [8, 9]. Because of the many advantages of NMA, scholars' interest in this method has gradually increased, and it has been widely used in the field of medicine and health [2, 10].

Bibliometric analysis is a statistical method of bibliographic counting that can assess and quantify the literature growth of a particular research content [11]. Bibliometric methods can extract and analyze the characteristics of publications including years, journals, authors, countries, and keywords to provide development trends or future research orientations of a specific subject [12, 13], which can help scholars grasp the development characteristics of the field and guide their future research [14]. In recent years, there have been a lot of studies in various fields that were published using this method. Li et al. [15] analyzed the international collaborations and academic relationships on haze research. Ruiz-Real et al. [16] identified the development trends and future research initiatives in the field of circular economy and environment. Gimenez-Espert and Prado-Gasco [17] analyzed the evolution of and current status of six nursing journals and presented the most cited articles, co-citations, and co-authors. Liang et al. [18] demonstrated the status quo, intellectual base, and hot topics in the field of medication literacy. There also has been a study published in 2015 that analyzed the global research collaboration of NMA using social network analysis methods [19]; however, this research did not analyze the co-cited journals**,** co-cited authors**,** co-cited references, and research hotspots. Besides, the number of NMAs has increased significantly in recent years, and global research cooperation may have changed. Therefore, it is necessary to analyze the current status of NMA research through bibliometric methods.

This study aimed to (1) analyze the distribution of publication outputs, journals, countries, institutes, authors, keywords, and references on NMA research; (2) identify the cooperation of countries and institutes; (3) and explore the development dynamics and existing hot topics.

## Methods

### Data source and search strategy

The Web of Science (WoS) Core Collection was retrieved to obtain relevant NMAs from inception to December 2018. The search strategy: TS = (“network meta analysis” OR “network meta analyses” OR “network meta-analysis” OR “network meta-analyses” OR “network metaanalyses” OR “network metaanalysis” OR “mixed treatment comparison meta analysis” OR “mixed treatment comparisons meta analyses” OR “mixed treatment meta analysis” OR “mixed treatment meta analyses” OR “mixed treatment comparison meta-analysis” OR “mixed treatment comparisons meta-analyses” OR “mixed treatment meta-analysis” OR “mixed treatment meta-analyses” OR “mixed treatment comparison metaanalysis” OR “mixed treatment comparisons metaanalyses” OR “mixed treatment metaanalysis” OR “mixed treatment metaanalyses” OR “multiple treatment comparison meta analysis” OR “multiple treatment comparisons meta analyses” OR “multiple treatments meta analysis” OR “multiple treatments meta analyses” OR “multiple treatment meta analysis” OR “multiple treatment meta analyses” OR “multiple treatment comparison meta-analysis” OR “multiple treatment comparisons meta-analyses” OR “multiple treatments meta-analysis” OR “multiple treatments meta-analyses” OR “multiple treatment meta-analysis” OR “multiple treatment meta-analyses” OR “multiple treatment comparison metaanalysis” OR “multiple treatment comparisons metaanalyses” OR “multiple treatments metaanalysis” OR “multiple treatments metaanalyses” OR “multiple treatment metaanalysis"); index: (SCI-EXPANDED). In the present study, only article and review papers were included. All searches were done within the same day to avoid the bias caused by the daily database updates. There were no restrictions on language, data category, and publication year.

### Statistical analysis

We used HistCite 2.1 (HistCite Software LLC, New York, USA) and CiteSpace V (Drexel University, Philadelphia, PA, USA) to analyze publication characteristics, including publication languages, years, journal sources, co-cited journals, countries, institutes, authors, co-cited authors, co-cited references, and keywords. Excel 2016 (Redmond, WA, USA) was used to analyze the publication trend. The three-term polynomial (Trinomial model) was applied to forecast the growth of publications in the following year [14]. We used CiteSpace V software to evaluate the relationships among the high-yield countries, institutes, and high-frequency keywords [20], and generate visual network maps for countries, institutions, and keywords. In the visual network maps, nodes represent the analytical characteristics, such as countries, institutes, and keywords, and the links between nodes reflect the co-operation, co-occurrence, or co-citation [21–24]. The size of the nodes reflects the number of publications or frequency, and the different colors of nodes and lines represent different times [25]. Purple circles represent centrality, and nodes with a larger centrality are often seen as key points in the network [18, 22]. We also performed cluster analysis for keywords and all clusters were named according to the terms extracted from the articles. Furthermore, we identified references with strong citation bursts through CiteSpace V.

The parameters of CiteSpace were as follows: time slicing (2002–2018), years per slice (1), term source (all selection), node type (choose one at a time), selection criteria (30), pruning (none), and visualization (cluster view-static, show merged network).

## Results

### Publication language

**Fig. 1:**
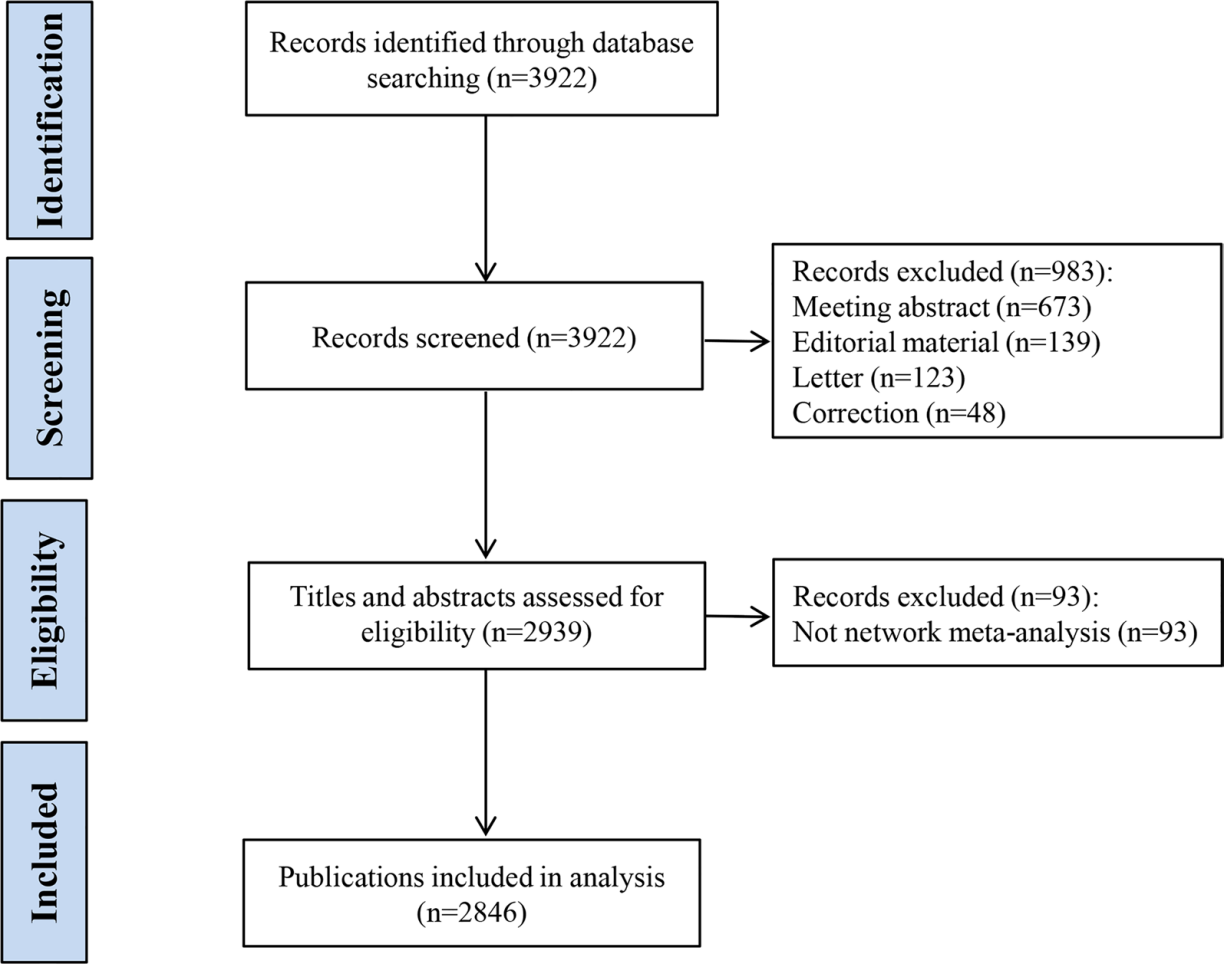
The flowchart of the screening process

A total of 2846 articles were included (Fig. 1), which were published in six languages. Among the 2846 articles, 2821 (99%) were published in English, 11 published in Spanish, 6 published in German, 5 published in French, 2 published in Polish, and 1 published in Russian.

### Publication outputs

**Fig. 2:**
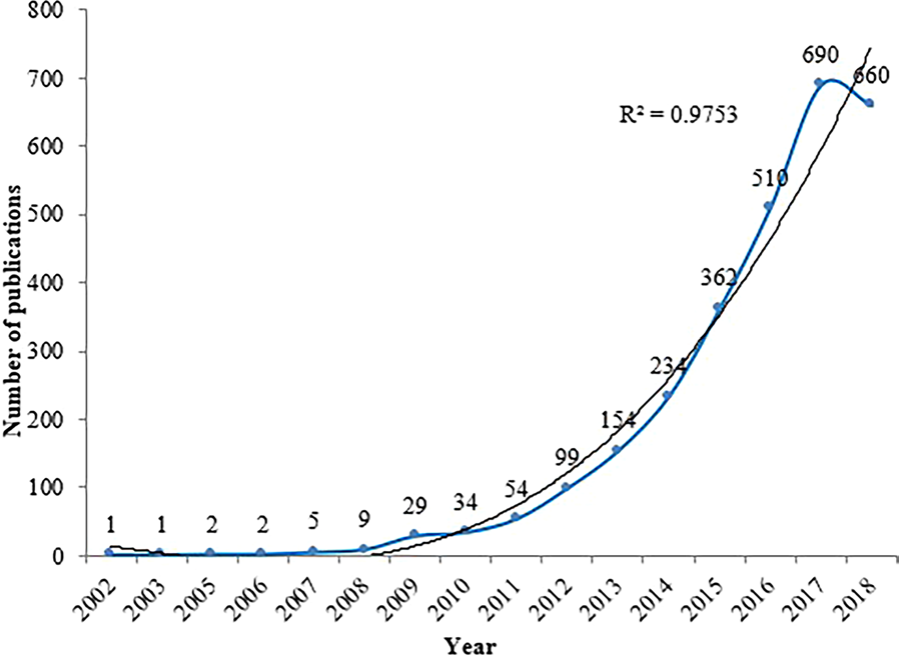
Publication years and growth forecast for NMA research

The first paper of included NMA research was published in 2002, and the NMA research developed gradually before 2010 and rapidly in the following years. As shown in Fig. 2, the number of publications per year was less than 10 before 2009. After 2010, the number of articles increased rapidly and broke through 200 articles in 2014, 500 articles in 2016, and 600 articles in 2017. Among them, the growth rate from 2015 to 2017 was the fastest and increased from 362 (13%) in 2015 to 690 (24%) in 2017. From 2013 to 2018, 2610 articles were published, accounting for 92% of all the included studies. The three-term polynomial model was used to evaluate the relationship between the number of publications and the year (excluding the data for 2018). It was found that the polynomial curve fits well with the annual literature growth trend with a high coefficient of determination (R^2^ = 0.9753). By the fitting curve, we can predict that the annual articles will continue to grow in the coming years.

### Journals and co-cited journals

In total, 771 journals published articles in NMA research. Table 1 presented the top 10 journals and co-cited journals in NMA research. The journal with the largest number of publications was *PLoS One* (110, 3.9%), followed by *Medicine* (85, 3.0%), *BMJ Open* (78, 2.7%), and *Cochrane Database Syst Rev* (68, 2.4%). Of the top 10 journals, three were from the United States (USA), seven from the United Kingdom (UK), and the impact factors of seven journals were lower than 4.500. *N Engl J Med* was the most co-cited journal, with 5904 co-citations, followed by *Lancet* (4888 co-citations), *Stat Med* (3696 co-citations), *J Am Coll Cardiol* (3101 co-citations), and *Circulation* (2666 co-citations). Among the top 10 co-cited journals, 50% are from the UK, and 50% from the USA, and seven journals with the impact factors higher than 15.000, three journals with the impact factors higher than 45.000.

**Table 1:** The top 10 journals and co-cited journals in NMA research [n (%)]

Rank	Journal	N (%)	Country	IF (2018)	Co-cited journal	Co-citation	Country	IF (2018)
1	PLoS One	110 (3.9%)	USA	2.766	N Engl J Med	5904	USA	70.670
2	Medicine	85 (3.0%)	USA	1.870	Lancet	4888	UK	59.102
3	BMJ Open	78 (2.7%)	UK	2.376	Stat Med	3696	UK	1.847
4	Cochrane Database Syst Rev	68 (2.4%)	UK	7.755	J Am Coll Cardiol	3101	USA	18.639
5	Oncotarget	63 (2.2%)	USA	Non-SCI	Circulation	2666	USA	23.054
6	Sci Rep	45 (1.6%)	UK	4.011	BMJ	2610	UK	27.604
7	Res Synth Methods	44 (1.6%)	UK	5.043	J Clin Epidemiol	2325	UK	4.650
8	BMJ	43 (1.5%)	UK	27.604	JAMA	2267	USA	51.273
9	Curr Med Res Opin	41 (1.4%)	UK	2.345	Cochrane Database Syst Rev	2029	UK	7.755
10	Stat Med	37 (1.3%)	UK	1.847	Ann Intern Med	1994	USA	19.315

**Fig. 3:**
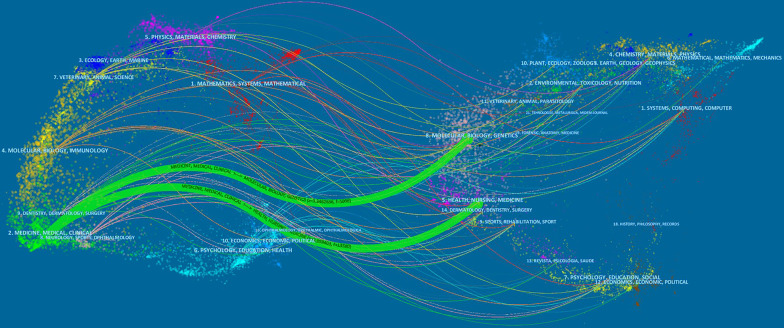
The dual-map overlay of journals related to NMA research

Figure 3 showed the dual-map overlay of journals. The yellow, green, and purple spline waves represent citations made by the source articles. Each spline curve starts with the citing map on the left and points to the cited map on the right. The label represented the subject covered by the journal [26]. In the current map, there were two main citation paths.

### Countries and institutes

**Fig. 4:**
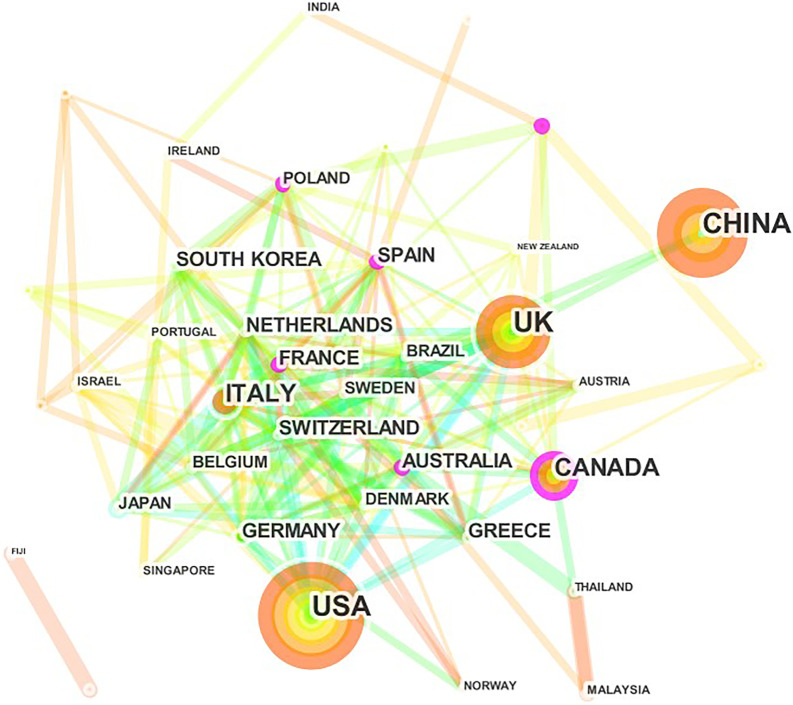
The network map of countries for NMA research

We reclassified articles from England, Scotland, Northern Ireland, and Wales to the UK and articles from Hong Kong, Macau, and Taiwan to China. In total, 85 countries involved in the publication of NMA research, and the USA (889, 31%) published the most articles, followed by China (740, 26%), UK (708, 25%), Canada (327, 11%), and the remaining countries published articles less than 300, Table 2. There were 43 nodes and 206 links in the network map of the country generated by CiteSpace (Fig. 4). The top three countries in terms of centrality (purple round) were Spain (0.19), Canada (0.17), and France (0.14). In general, the cooperation between developed countries was relatively close.

**Table 2:** The top 10 countries and institutes contributed to publications in NMA research [n (%)]

Rank	Country	N (%)	Institute	N (%)
1	USA	889 (31%)	University of Bristol (UK)	113 (4.0%)
2	China	740 (26%)	University of Toronto (Canada)	106 (3.7%)
3	UK	708 (25%)	McMaster University (Canada)	95 (3.3%)
4	Canada	327 (11%)	University of Ioannina (Greece)	81 (2.9%)
5	Italy	286 (10%)	University of Oxford (UK)	70 (2.5%)
6	Germany	228 (8.0%)	Columbia University (USA)	68 (2.5%)
7	Switzerland	193 (6.8%)	University of York (UK)	63 (2.2%)
8	Netherlands	192 (6.8%)	University of Ottawa (Canada)	61 (2.1%)
9	France	151 (5.3%)	Mayo Clinic (USA)	60 (2.1%)
10	Greece	122 (4.3%)	University of Bern (Switzerland)	57 (2.0%)

**Fig. 5:**
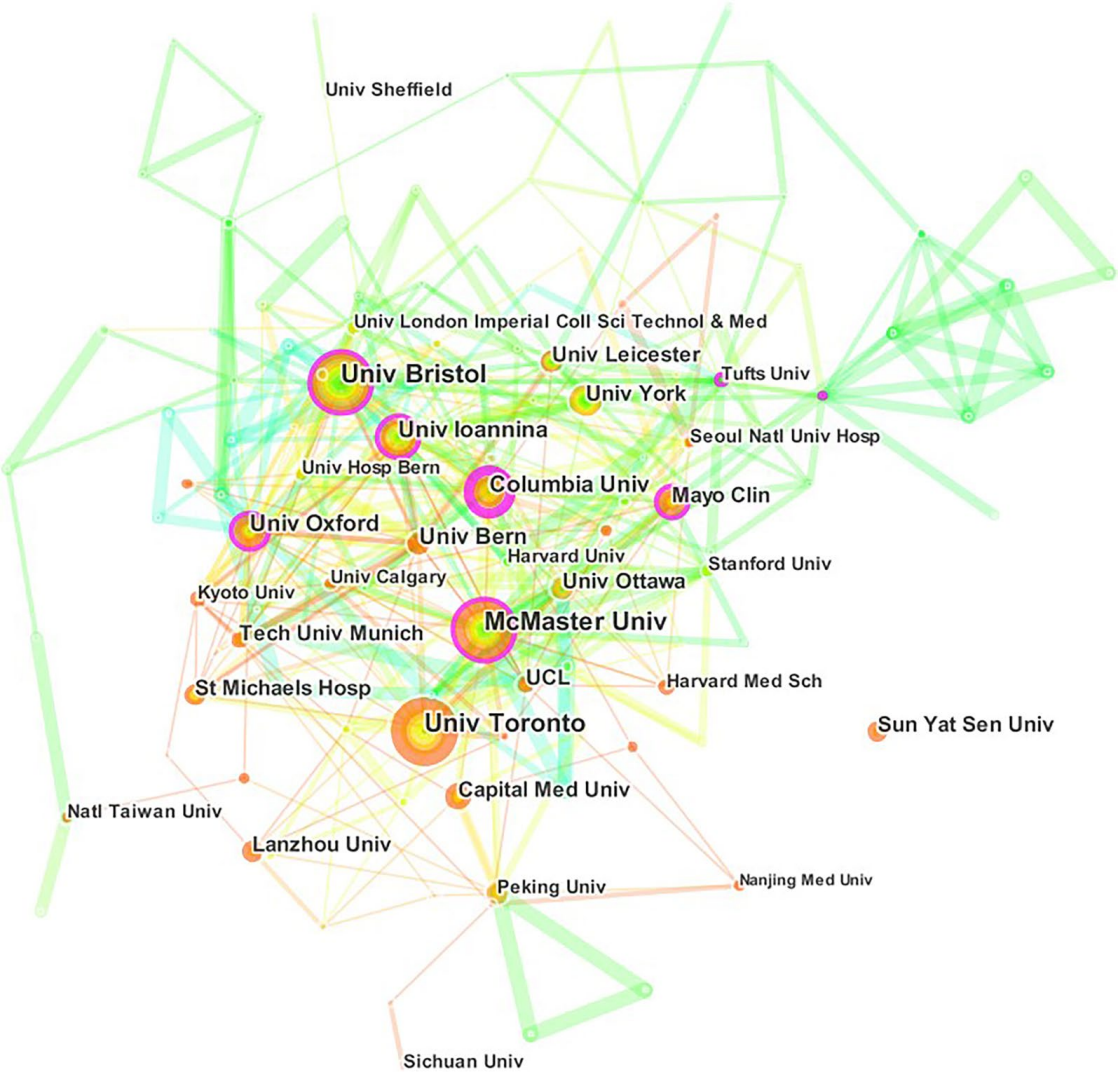
The network map of institutes for NMA research

3534 institutes contributed to the publications of NMA research. According to Table 2, the top five institutes were University of Bristol(113, 4.0%), University of Toronto (106, 3.7%), McMaster University (95, 3.3%), University of Ioannina (81, 2.9%), and University of Oxford (70, 2.5%). There were 145 nodes and 467 links in the network map of the institutes generated by CiteSpace (Fig. 5). The top five institutes in terms of centrality (purple round) were Columbia University (0.23), University of Bristol (0.20), University of Ioannina (0.16), McMaster University (0.15), and University of Oxford (0.15). In general, the cooperation between institutes was relatively close.

### Authors and co-cited authors

A total of 11,528 authors contributed to the NMA articles. Table 3 showed the top 10 authors and co-cited authors. Salanti G (67, 2.4%) from the University of Bern ranked first, followed by Welton NJ (47, 1.7%) and Dias S (40, 1.4%) from the University of Bristol, and Stone GW from the Columbia University Medical Center (37, 1.3%). The top five co-cited authors were Higgins JPT from University of Bristol (1670 citations), Salanti G from University of Bern (1450 citations), Dias S from University of Bristol (1279 citations), Lu G from University of Bristol (672 citations), and Jansen JP from Tufts University School of Medicine (662 citations). The remaining authors were cited times less than 600.

**Table 3:** The top 10 authors and co-cited authors in NMA research [n (%)]

Rank	Author	N (%)	Co-cited Author	Citations
1	Salanti G (University of Bern, Switzerland)	67 (2.4%)	Higgins JPT (University of Bristol, UK)	1670
2	Welton NJ (University of Bristol, UK)	47 (1.7%)	Salanti G (University of Bern, Switzerland)	1450
3	Dias S (University of Bristol, UK)	40 (1.4%)	Dias S (University of Bristol, UK)	1279
4	Stone GW (Columbia University Medical Center, USA)	37 (1.3%)	Lu G (University of Bristol, UK)	672
5	Ades AE (University of Bristol, UK)	36 (1.3%)	Jansen JP (Tufts University School of Medicine, USA)	662
6	Jansen JP (Tufts University School of Medicine, USA)	36 (1.3%)	Moher D (Ottawa Hospital Research Institute, Canada)	563
7	Biondi-Zoccai G (Sapienza University of Rome, Italy)	35 (1.2%)	Caldwell DM (University of Bristol, UK)	530
8	Tu YK (National Taiwan University, China Taiwan)	34 (1.2%)	Palmerini T (University of Bologna, Italy)	473
9	Cipriani A (University of Oxford, UK)	33 (1.2%)	White IR (MRC Clinical Trials Unit at UCL, UK)	420
10	Mavridis D (University of Ioannina, Greece)	32 (1.1%)	Cipriani A (University of Oxford, UK)	406

### Co-cited references and references with citation bursts

**Fig. 6:**
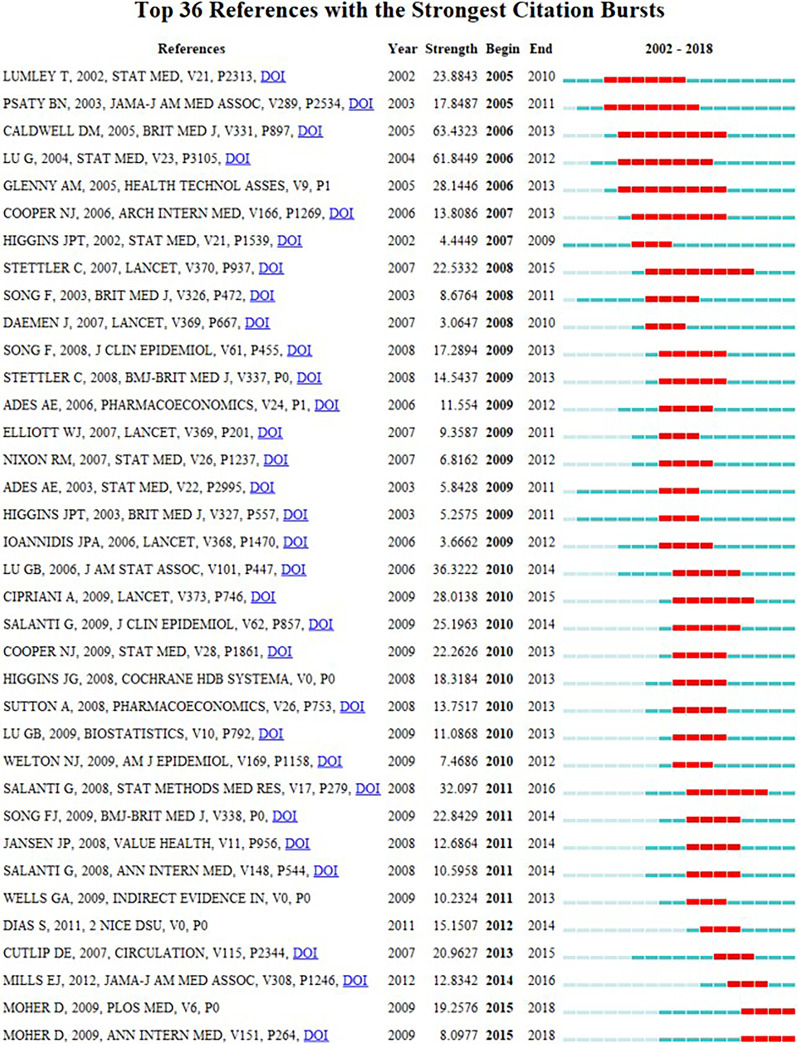
Top 36 references with strong citation bursts. *Note* The red bars mean some references cited frequently; the green bars were references cited infrequently

References with citation bursts are de-fined as those that are cited frequently over a period of time [23]. Table 4 revealed the top 10 co-cited references related to NMA research. Among them, the reference conducted by Lu and Ades [5] (619 co-citations) had the highest co-cited times, followed by the articles performed by Salanti et al. [27] (542 co-citations) and Caldwell et al. [8] (441 co-citations), the remaining seven references [1, 3, 28–32] were co-cited between 300 and 380 times. Figure 6 presented the top 36 references with strong citation bursts. The first reference [1] with citation bursts appeared in 2005, and 58% of the references appeared citation bursts between 2009 and 2011.

**Table 4:** Top 10 co-cited references related to NMA research

Rank	Co-cited reference	Co-citation
1	Lu G, 2004, STAT MED, V23, P3105 [5]	619
2	Salanti G, 2011, J CLIN EPIDEMIOL, V64, P163 [27]	542
3	Caldwell DM, 2005, BRIT MED J, V331, P897 [8]	441
4	Dias S, 2010, STAT MED, V29, P932 [28]	380
5	Higgins JPT, 2011, BMJ-BRIT MED J, V343 [29]	351
6	Chaimani A, 2013, PLOS ONE, V8 [3]	350
7	Salanti G, 2008, STAT METHODS MED RES, V17, P279 [30]	341
8	Lumley T, 2002, STAT MED, V21, P2313 [1]	334
9	Hutton B, 2015, ANN INTERN MED, V162, P777 [31]	321
10	Higgins JPT, 2003, BRIT MED J, V327, P557 [32]	308

### Co-occurrence keywords and cluster analysis

A total of 10,980 keywords were identified, but 7398 (67%) keywords appeared only one time. The keyword with the highest frequency was network meta-analysis (1424, 4.0%), followed by randomized controlled trials (816, 2.3%), double-blind (547, 1.6%), meta-analysis (463, 1.3%), and efficacy (419, 1.2%). The other keywords appeared less than 400 times.

**Fig. 7:**
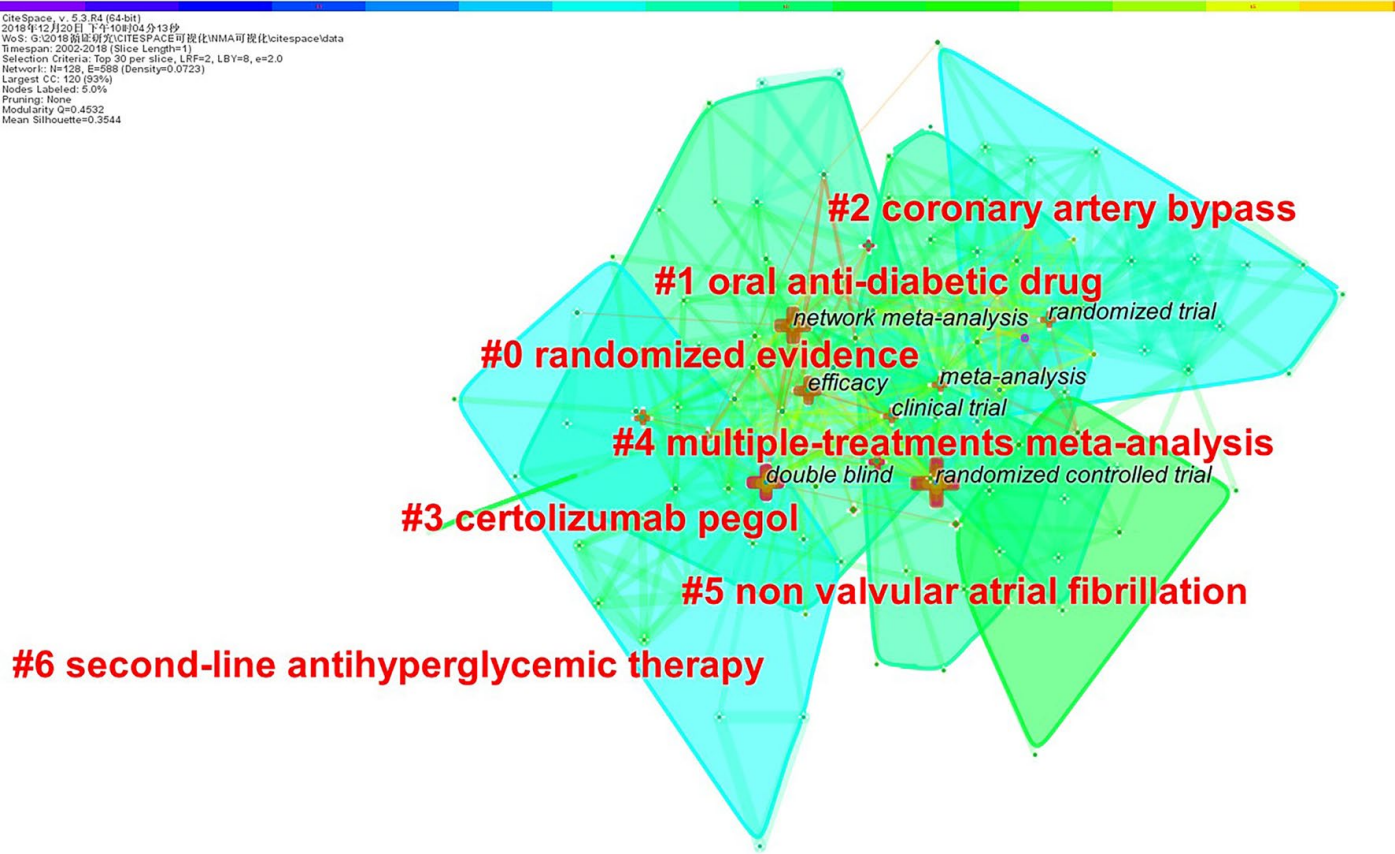
The network map of keywords for NMA research

Generating a keyword network map resulted in 128 nodes and 588 links (Fig. 7). All clusters were named after terms extracted from the included articles. In total, 7 clusters were identified. But cluster 4 named “multiple-treatments meta-analysis” is the keyword we use when performing the search and should be excluded. The remaining 6 clusters were named “#0 randomized evidence”, “#1 oral anti-diabetic drug”, “#2 coronary artery bypass”, “#3 certolizumab pegol”, “#5 non valvular atrial fibrillation”, and “#6s-line antihyperglycemic therapy”.

## Discussion

We conducted a literature search in the Web of Science and included 2846 articles, which were published between 2002 and 2018. Before 2009, only twenty articles were published in total and the number of publications per year was less than ten, indicating that NMA research developed slowly during this period. After 2010, the annual number of publications increased rapidly, especially between 2015 and 2017. The possible reasons for the dramatic increase might be the methodology of NMA has been greatly improved; countries or regions paid more attention to this area and increased financial support; more and more people have mastered the methods of conducting NMA and started to engage in related research. More than 99% of the included NMA studies were published in English, which is related to the database mainly containing English journal articles. This may be a barrier for researchers and medical professionals who do not speak English (fluently) in accessing and/or putting out NMA publications.

2846 articles published in 771 journals, but 12% of journals published more than five papers, 68% of journals published only one paper or two papers, revealing that many journals have contributed to the publication of NMA research, but only a few journals insisted on publishing relevant research. Of the top 10 journals, seven were from the UK, and the impact factors of seven journals were lower than 4.500, indicating the journals from the UK are more often publish NMA studies, but the impact factors of these high-yield journals are not high. Of the top 10 co-cited journals, only three are also top 10 productive journals, but 70% of journals have an impact factor greater than 15.000, which shows that the articles published in the high-impact factor journals are more often cited. Possible reason for this phenomenon may be that the high-impact journals have higher requirements on the topic selection and quality of published articles, moreover, it has higher social media dissemination and more likely to attract attention.

2846 articles involved in 3534 institutes in 85 counties. 82% of papers were published by the USA, China, and the UK, revealing that these three countries play a major role in promoting the development of NMA research. In general, the cooperation between developed countries was relatively close, but China has less cooperation with other countries, even though China had the second largest number of publications. Therefore, China should strengthen cooperation with other countries to improve the quality and influence of NMA research. The top 10 institutes contributed to 774 papers, which accounted for 27% of the included studies. Of the top 10 institutes, 3 were from the UK, 3 from Canada, 2 from the USA, and 1 each from Greece, and Switzerland. It is worth mentioning that there were no institutes from China, Italy, Germany, Netherlands, and France among the top 10 institutes. The active collaborations were observed between the main institutes, especially between institutes from the same country.

The dynamics of a field can be characterized in part by articles with citation bursts [33]. The first reference that was detected with citation burst is the study conducted by Lumley in 2002 [1], and the burst started in 2005 and ended in 2010. This article provided a new method for indirect comparisons. Among citation bursts starting in 2009, the strongest burst is due to a 2008 paper by Song et al. [34] This study assessed the discrepancies between direct and adjusted indirect comparisons of new versus conventional pharmaceutical interventions and showed that adjusted indirect comparisons can be used to test the validity and applicability of results from head-to-head randomized trials [34], which drives the development of indirect comparisons. The strongest burst starting from 2010 is associated with a 2006 paper by Lu and Ades [35] on evidence inconsistency in mixed treatment comparisons. The authors proposed a new method for assessing evidence inconsistency in the framework of Bayesian hierarchical models. Citation bursts starting in 2011 are led by Salanti et al.'s [30] article published in 2008. This methodological article focused on an important element of the NMA: network. The authors introduced the concepts of network geometry and asymmetry and proposed some methods to deal with the extent of network asymmetry [30]. The most recent strongest burst started in 2015 is associated with Moher et al. in 2009 [36]. This article presented a new reporting tool for systematic reviews called PRISMA (Preferred Reporting Items for Systematic Reviews and Meta-analyses). Subsequently, Hutton et al. [31] proposed the PRISMA extension statement based on the PRISMA in 2015, which is now widely used to assess the reporting quality of NMA.

Hotspots are scientific questions or topics discussed in a set of documents that are intrinsically linked to a certain period of time [18]. In bibliometrics, a network graph of keyword co-occurrences can reflect hot topics [22]. In the current study, we used CiteSpace V to conducted cluster analysis for keywords, and the results showed that there are 7 clusters in the field of NMA. Cluster #0 is the largest cluster and contains 38 keywords. This cluster mainly focused on randomized evidence. Randomized controlled trials (RCTs) are considered to be the most reliable source of information on relevant treatment outcomes [37] and NMA usually only includes evidence from RCTs. When non-randomized evidence is included in the NMA, the transmission and consistency of the study are amplified, so the results may not be very accurate [38]. But in recent years, more and more NMAs have included non-randomized evidence [39, 40]. However, randomized evidence remains the main source of evidence for NMA. Cluster #1 includes 25 keywords and focuses on the effects of oral anti-diabetic drugs on diabetes. Cluster #6 focuses on second-line antihyperglycemic therapy. It is estimated that more than 415 million adults worldwide have diabetes and the prevalence is increasing, with more than 640 million adults expected to have diabetes by 2040 [41, 42]. The risk of adverse effects of cardiovascular disease, heart failure, and kidney disease in diabetic patients is increasing [43–45], and the age of death of patients is decreasing [46, 47], which has attracted widespread attention among people in the medical and health fields. Therefore, the efficacy and adverse outcomes of various measures used for the treatment of diabetes have become one of the hot topics in NMAs. Cluster #2 focuses on coronary artery bypass, one of the most common cardiac procedures in the world, and is the gold standard treatment for most multivessel coronary and left main coronary artery disease [48, 49]. Cluster #3 mainly related to certolizumab pegol, a tumor necrosis factor blocker, can be used for the treatment of rheumatoid arthritis, Crohn's disease, psoriatic arthritis, and axial spondyloarthritis [50, 51]. Cluster #5 focuses on non-valvular atrial fibrillation, a type of atrial fibrillation that occurs without a mechanical prosthetic heart valve and moderate to severe mitral stenosis [52]. In general, the main hot topics covered by NMA are the treatment of diabetes, cardiovascular diseases, and immune rheumatism.

### Strengths and limitations

As far as we know, this is the first study to perform a bibliometric analysis of NMA research by using CiteSpace and HistCite. In addition to analyzing the social network relationships of institutions and countries, we also carried out cluster analysis of keywords and detection of burst references, which can clearly show the hot topics and development trends of NMA. But our study also has some limitations. First, we only included the NMA articles published in the WoS database, which may not fully reflect the current status of all NMA research, although WoS is considered to be the most important data source for bibliometric analysis in science [11]. Second, almost all of the included studies are in English, which may lead to selection bias. Therefore, the results may not be applicable to NMAs published in other languages [19]. Third, there are some inconsistencies in the data analysis process, such as one author from different units, one organization with different names, and the same meaning keywords have different expressions. Although we have standardized the authors, institutions, and keywords in our research, potential errors may still exist.

## Conclusions

NMA studies have significantly increased over the past decade, especially from 2015 to 2017. Worldwide, researchers in the field come mostly from Western Europe and North America, mainly spread in the USA, China, and UK. There were active collaborations between developed countries, it is suggested that cooperation should be strengthened between developed countries and developing counties in the future. The University of Bristol, University of Toronto, and McMaster University were the top three most productive institutes. The active collaborations were observed between the main institutes, especially between institutes from the same country. There were seven hot topics. The treatment of diabetes, cardiovascular diseases, and immune rheumatism may be the main hot topics.

## Data Availability

All data generated or analyzed during this study are included in this published article.

## Abbreviations

NMA: Network meta-analysis
RCTs: Randomized controlled trials
